# Congenital Epulis of the Newborn: A Report on Two Cases

**DOI:** 10.7759/cureus.25730

**Published:** 2022-06-07

**Authors:** Bader AlAllah, Jubara Alallah, Farzeen Mohtisham

**Affiliations:** 1 College of Medicine, King Saud Bin Abdulaziz University for Health Sciences, Jeddah, SAU; 2 Pediatrics, Ministry of the National Guard-Health Affairs, King Abdulaziz Medical City, Jeddah, SAU; 3 Neonatology, King Abdullah International Medical Research Center, Jeddah, SAU; 4 Neonatology, King Abdulaziz Medical City, Riyadh, SAU

**Keywords:** newborn health, tumor´, congenital epulis, epulis, congenital

## Abstract

Granular cell epulis is a rare benign tumor of the newborn. It originates from the alveolar ridge, most commonly from the maxillary alveolar ridge. Despite its striking appearance, the lesion is ultimately benign. However, immediate surgical treatment is required if there is a risk of airway obstruction or feeding difficulties. We report two cases of granular cell epulis presented at birth, the first with a large mass originating from the maxillary alveolar ridge and the second with the mass originating from the mandibular alveolar ridge. Both were successfully managed with surgical excision without complications. Histopathology of both masses confirmed the granular cell epulis diagnosis.

## Introduction

Granular cell epulis is a rare tumor of the newborn and is also known as granular cell tumor of the newborn and Neumann’s tumor. This lesion on the gingiva was called “epulis” after the Greek for “to boil the gums” or “on the gums.” Neumann was the first to depict gingival overgrowth in 1871 [1]. Over 200 cases have been reported in the literature [2]. The tumor develops from the gingiva mucosa, most usually in the anterior region of the maxillary alveolar ridge. It manifests as a lump protruding from the newborn’s mouth, obstructing breathing or feeding if larger in size. Granular cell epulis is only observed in newborns and is unlike other granular cell tumors [3]. The tumor has a substantial 8:1 female prevalence. It usually appears as a single mass, although numerous masses may occur in 10% of cases [4]. Prompt surgical resection is the preferred treatment. There have been no reports of tumor recurrence or damage to future dentition, implying that radical excision is unnecessary [5]. Our two cases presented at birth with a similar clinical presentation; the same management was applied in both cases. This article will raise awareness of this uncommon condition and how to treat it.

## Case presentation

Patient 1

**Figure 1:**
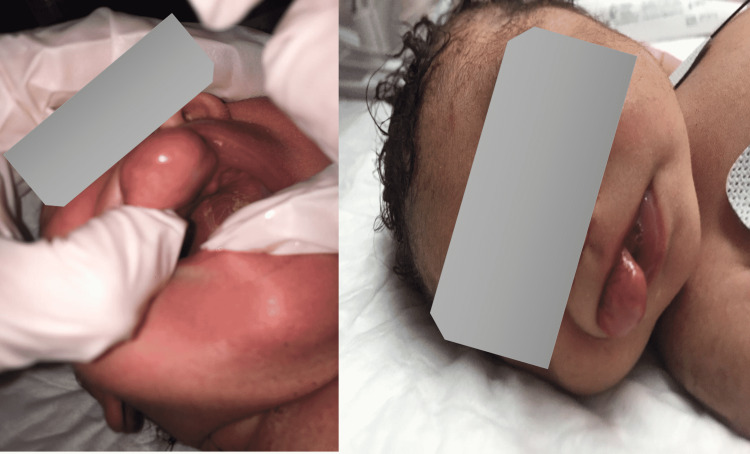
Case 1: A pedunculated mass originating from the upper gingiva, around 3 x 2 x 3 cm, was soft, pink, and firm in consistency.

A full-term female infant was born to a gravida 4 para 1 + 2 mother at 40 weeks. At birth, she was noted to have a bulky mass originating from the upper gingiva protruding from the mouth immediately after delivery (Figure 1). The mother was booked into a tertiary hospital and had regular antenatal visits. A fetal ultrasound (US) had been carried out twice; both were normal, with the second US carried out at 29 weeks. The mother went into labor and was delivered by emergency cesarean section due to pathological cardiotocography (CTG). The baby was active and vigorous at birth. The Apgar score was 9 at one minute and five minutes; the cord pH was 7.21, birth weight was 3280 g, length was 51 cm, and head circumference was 34.5 cm. The baby was appropriate for gestational age.

She was otherwise active, with no dysmorphic features, and had no other anomalies. Her breathing was normal, with no distress or desaturation, and she remained hemodynamically stable. Routine lab tests were normal. Maxillofacial surgery was consulted, and they decided to perform surgical resection. Excision of the mass was undertaken on day 3 of life. Post-operatively, she was extubated immediately, and the following day she was started on oral feeding and was breastfeeding well. She was discharged home in good condition, and there was no evidence of recurrence at a follow-up clinic visit.

Patient 2

**Figure 2:**
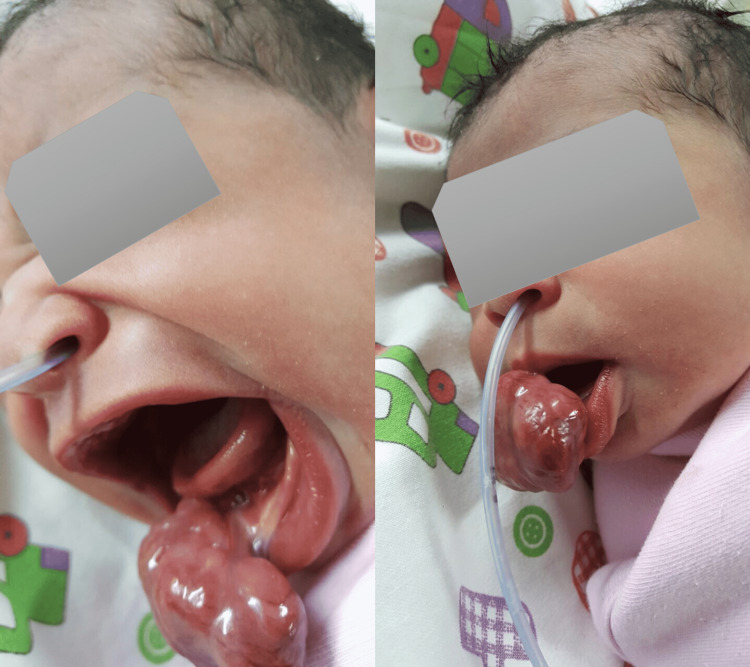
Case 2: A pedunculated mass originating from the lower gingiva, around 5 x 4 x 3 cm, was lobulated and firm in consistency.

The second case is a full-term female, a product of spontaneous vaginal delivery to a consanguineous parent with normal antenatal follow-up. All fetal US were reported as normal. Her Apgar score was 9 at both one and five minutes, cord pH was 7.35, birth weight 3400 g, length 52 cm, and head circumference 35 cm. She was appropriate for gestational age. She was noted to have a pedunculated mass originating from the lower gingiva, around 5 x 4 x 3 cm in size, firm in consistency, and lobulated (Figure 2). She was otherwise very active, with no dysmorphic features, and no other anomalies were detected. Her breathing, hemodynamic, and laboratory blood tests were normal.

Similar to case 1, excision of the mass was undertaken on day 2 of life, with no intraoperative complications. She was discharged home in a stable condition 48 hours later on full feeding. She was seen in the follow-up clinic with no evidence of recurrence.

The mass biopsy of both patients revealed a circumscribed lesion lined by stratified squamous epithelium. The submucosa contained sheets of large polygonal cells with small central round nuclei and abundant eosinophilic granular cytoplasm, and the pathological diagnosis confirmed the preoperative diagnosis of granular cell epulis.

## Discussion

Granular cell epulis is the most commonly used and recognized word for a rare neonatal benign tumor that is distinct from other granular cell tumors. Granular cell epulis appears as a lump in the mouth that originates from the alveolar ridge. It is more common in females than males and originates in the maxillary alveolar ridge. It displays no signs of malignancy or recurrence [3]. The tumor is usually a single mass, but numerous or lobulated tumors are also rarely described. Depending on the size of the mass, they may affect breathing or oral feeding. Antenatal diagnoses of granular cell epulis have been reportedly illustrated through the fetal US as early as 26 weeks of gestation [6]. The antenatal scan of both patients considered in this study was reported to be normal. It is likely that the granular cell epulis arose in the third trimester, and it typically comes as a surprise to parents and healthcare providers in the delivery room. Usually, the mass does not enlarge after birth, the mass is the same color throughout, and it is a painless lesion. The smaller the lesions, the higher chance of spontaneous regression later.

In histopathology, large, round, and polyhedral histiocyte-like cells with small, oval-to-round nuclei and copious eosinophilic granular cytoplasm make up the bulk of the mass. The intervention of granular cell epulis depends on the size. The larger the size, which may interfere with feeding and breathing, necessitates surgical excision. Nonsurgical intervention is better for small masses as spontaneous regression may occur.

Because granular cell epulis appears in newborns, the maternal hormonal impact may play a role in the progression of the lesion [3]. Whether granular cell epulis is a true neoplastic or reactive lesion is unknown. However, a non-neoplastic origin is supported by the chance of spontaneous regression in some cases, the lack of recurrence after surgical removal, and the lack of a malignant counterpart [7].

## Conclusions

Granular cell epulis is a rare soft-tissue tumor that arises from the oral cavity. Intervention depends on the size, as it may interfere with feeding and breathing. Most cases of congenital epulis can be diagnosed clinically. Radiological investigation postnatally may not be needed for uncomplicated cases. The overall prognosis of granular cell epulis is good.

## References

[1] Neumann E (1871). Ein fall von kongenitaler epulis. Arch Heilkd.

[2] Ritwik P, Brannon RB, Musselman RJ (2010). Spontaneous regression of congenital epulis: a case report and review of the literature. J Med Case Rep.

[3] Lapid O, Shaco-Levy R, Krieger Y, Kachko L, Sagi A (2001). Congenital epulis. Pediatrics.

[4] Kanotra S, Kanotra SP, Paul J (2006). Congenital epulis. J Laryngol Otol.

[5] Kumar RM, Bavle RM, Umashankar DN, Sharma R (2015). Congenital epulis of the newborn. J Oral Maxillofac Pathol.

[6] Rehman MU, Khanani MF, Bekdache G, Rehman Durrani NU, Jamil A, Rahmani A, Chedid F (2012). Congenital epulis. J Coll Physicians Surg Pak.

[7] Zarbo RJ, Lloyd RV, Beals TF, McClatchey KD (1983). Congenital gingival granular cell tumor with smooth muscle cytodifferentiation. Oral Surg Oral Med Oral Pathol.

